# Supplementary material for: Evidence supported by Mendelian randomization: impact on inflammatory factors in knee osteoarthritis
Source: Front Med (Lausanne). 2024 May 28;11:1382836. doi: 10.3389/fmed.2024.1382836 (PMC11165061; doi:10.3389/fmed.2024.1382836)
Supplement: Supplementary file 1 [file Data_Sheet_1.ZIP › data/GCST90274830_5e6/830.forest.pdf]

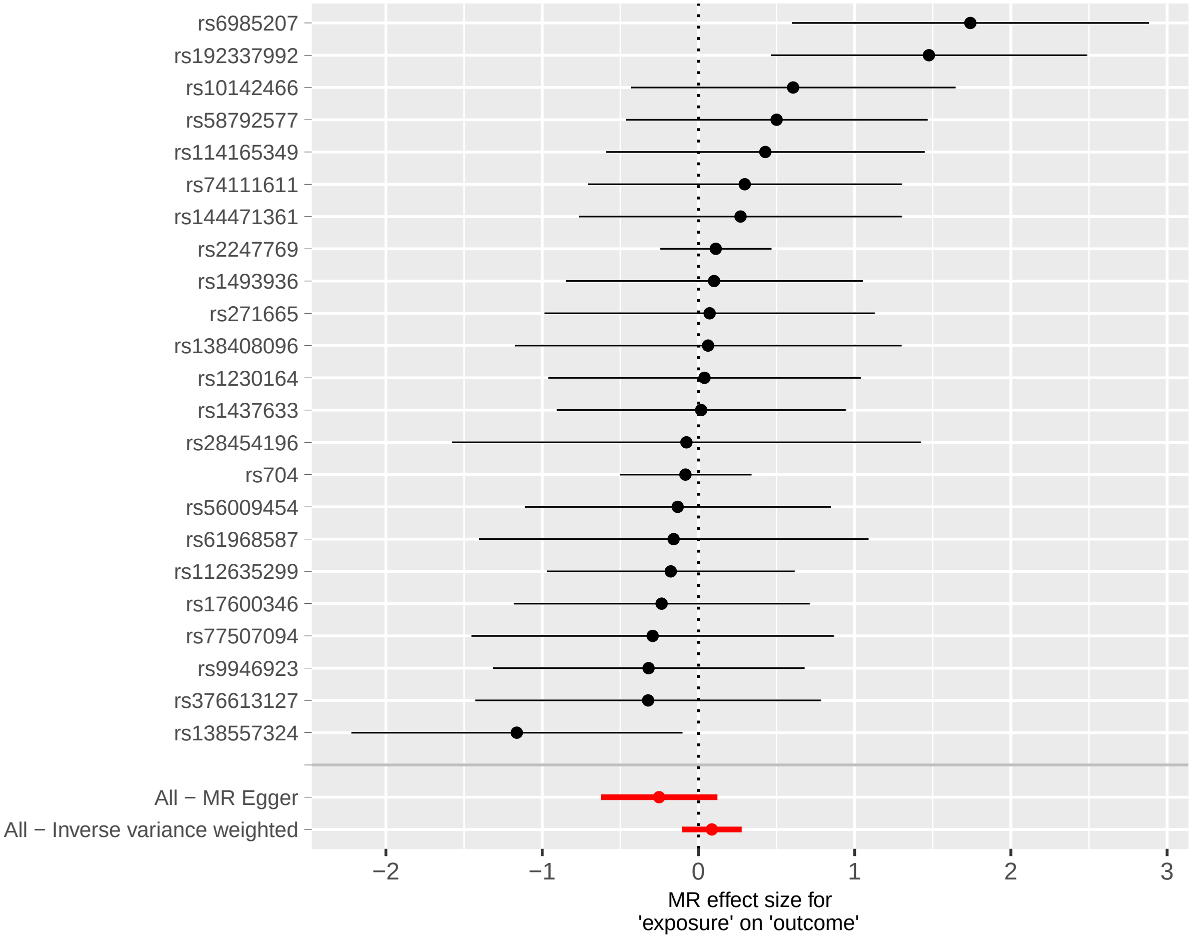
rs6985207
rs192337992
rs10142466
rs58792577
rs114165349
rs74111611
rs144471361
rs2247769
rs1493936
rs271665
rs138408096
rs1230164
rs1437633
rs28454196
rs704
rs56009454
rs61968587
rs112635299
rs17600346
rs77507094
rs9946923
rs376613127
rs138557324
All − MR Egger
All − Inverse variance weighted
−2
−1
0
1
2
3
MR effect size for
'exposure' on 'outcome'
